# Quantification of the myocardial partition coefficient for intravenous iron (ferric carboxymaltose) using T1 mapping cardiovascular magnetic resonance

**DOI:** 10.1186/1532-429X-18-S1-P229

**Published:** 2016-01-27

**Authors:** Goran Abdula, Arman Valadkhani, Magnus Lundin, Peder Sörensson, Andreas Sigfridsson, Martin Ugander

**Affiliations:** 1 Department of cardiology, Karolinska University Hospital, Karolinska Institute, Stockholm, Sweden; 2 Department of clinical physiology, Karolinska University Hospital, Karolinska Institute, Stockholm, Sweden

## Background

Cardiovascular magnetic resonance (CMR) T1 mapping has recently been shown to be highly sensitive for detecting myocardial iron overload. Furthermore, T1-mapping can be used to quantify relative concentrations of contrast agents that shorten T1. We hypothesized that T1 mapping can detect and quantify the myocardial distribution of a clinically available intravenous iron substitution agent.

## Methods

CMR imaging was performed in healthy male volunteers (n = 8, mean ± SD age 27 ± 3 years). T1 of blood and myocardium was quantified using a modified Look-Locker inversion recovery (MOLLI) sequence for T1-mapping in a mid-ventricular short-axis slice at 1.5T (Siemens Aera). Images were acquired before and at regular intervals up to 50 minutes after onset of a 15 minute long injection of 20 ml (50 mg iron/ml) ferric carboxymaltose (Vifor Pharma). T1, R1 and ΔR1 of myocardium and blood, and the partition coefficient (lambda) for myocardium were measured over time.

## Results

**Figure 1:**
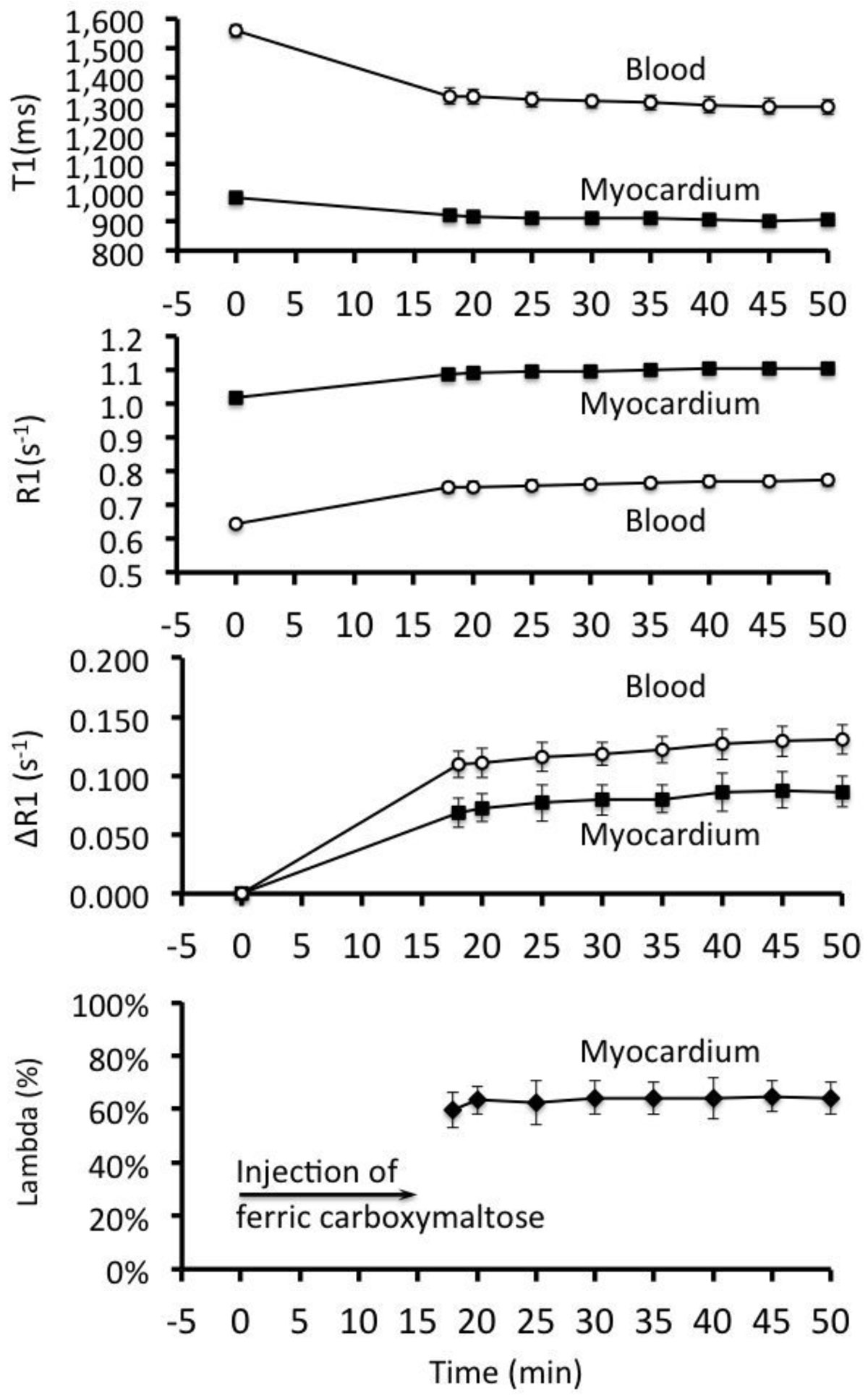
**Mean values (± SEM) for myocardial (black squares) and blood (white circles) T1, R1, ΔR1 and partition coefficient (lambda) over time after injection of ferric carboxymaltose in healthy volunteers (n = 8)**. R1 is equal to 1/T1. ΔR1 is the difference between post-contrast and pre-contrast R1 and is proportional to contrast concentration. Lambda is the ratio of ΔR1 of the myocardium to ΔR1 of the blood. Note that lambda is effectively unchanged between 20-50 minutes after injection.

Both myocardial and blood T1 were shortened after intravenous injection of ferric carboxymaltose (Figure 1). Thus, there is an increase in both R1, which is the reciprocal of T1, and ΔR1, which is the difference between post-contrast and pre-contrast R1, and is linearly related to contrast concentration. Notably, lambda, which is the ratio of ΔR1 of myocardium to ΔR1 of blood, remains constant over time (mean ± SEM, 61 ± 6% at 30 minutes).

## Conclusions

**Figure 2:**
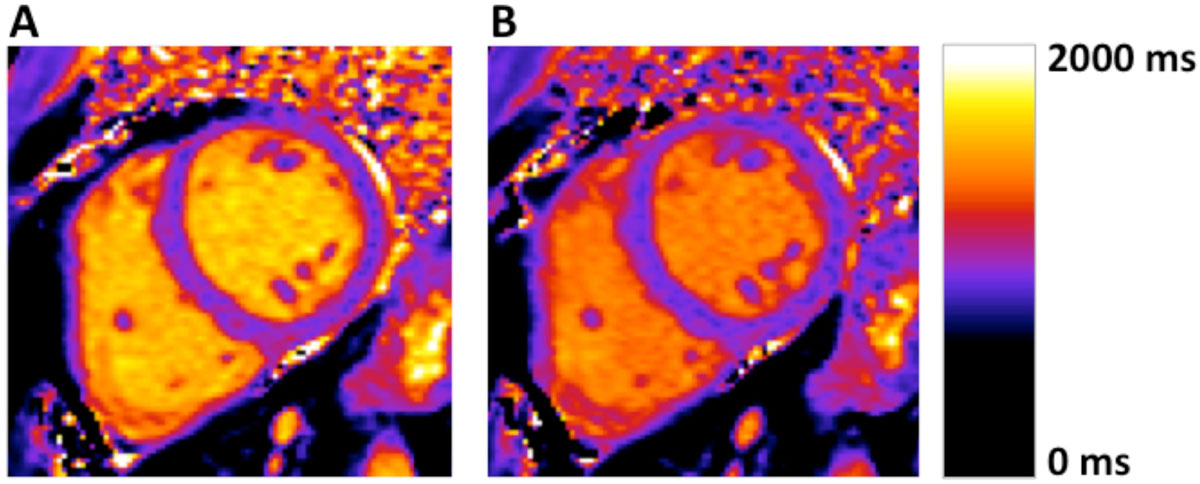
**(A) Pre-contrast (native T1) and (B) post-contrast (25 minutes after onset of ferric carboxymaltose injection) short-axis Modified Look-Locker Inversion recovery (MOLLI) sequence in a mid-ventricular slice**. Note how T1 is visibly shorter following injection of ferric carboxymaltose compared to native T1

T1 mapping can be used to detect and quantify the myocardial distribution of ferric carboxymaltose (figure 2). Furthermore, since lambda remains unchanged over time, the concentration of ferric carboxymaltose in normal myocardium is in a dynamic equilibrium with the blood pool 20-50 minutes after intravenous injection. However lambda in healthy myocardium for ferric carboxymaltose (~60%) was considerably higher than lambda for gadolinium-based extracellular contrast agents from the literature (~40-45%)^1^, thus indicating that ferric carboxymaltose distributes to a greater extent into the myocardium than extracellular agents, most likely by distribution also into the intracellular space. The described technique opens up the new possibility for using CMR to study *in vivo* myocardial iron physiology in health and disease.

